# The Search for Biomarkers in Fibromyalgia

**DOI:** 10.3390/diagnostics11020156

**Published:** 2021-01-21

**Authors:** Kevin V. Hackshaw

**Affiliations:** Department of Internal Medicine, Division of Rheumatology, Dell Medical School at the University of Texas at Austin, 1601 Trinity St, Austin, TX 78712, USA; Kevin.Hackshaw@austin.utexas.edu

**Keywords:** fibromyalgia, vibrational spectroscopy, biomarker, cytokines, central sensitization

## Abstract

Fibromyalgia is the most common of the central sensitivity syndromes affecting 2–5% of the adult population in the United States. This pain amplification syndrome has enormous societal impact as measured by work absenteeism, decreased work productivity, disability and injury compensation and over-utilization of healthcare resources. Multiple studies have shown that early diagnosis of this condition can improve patient outlook and redirect valuable healthcare resources towards more appropriate targeted therapy. Efforts have been made towards improving diagnostic accuracy through updated criteria. The search for biomarkers for diagnosis and verification of Fibromyalgia is an ongoing process. Inadequacies with current diagnostic criteria for this condition have fueled these efforts for identification of a reproducible marker that can verify this disease in a highly sensitive, specific and reproducible manner. This review focuses on areas of research for biomarkers in fibromyalgia and suggests that future efforts might benefit from approaches that utilize arrays of biomarkers to identify this disorder that presents with a diverse clinical phenotype.

## 1. Introduction

The National Institutes of Health (NIH) defines biomarkers as: “characteristics that can be objectively measured and evaluated as an indication of normal or pathogenic processes or pharmacological responses to a therapeutic intervention“ [1]. Studies that show alterations between diseased and healthy states have discovered differentiating metabolites. An independent study that replicates and validates the prior study helps to classify the differentiating metabolites as a true biomarker [2]. Therefore, taking these definitions and criteria into consideration, it is evident that Fibromyalgia (FM) is a condition with no known biomarkers.

## 2. Background

Some of the most common and difficult to treat disorders in medicine are the “medically unexplained symptoms” [3,4] or “central sensitivity syndromes” (CSS) [3,4,5] including FM. Approximately 2% of the adult population in the United States are affected by FM, including 3.9% of women ages 20–40 and 5.8% of women ages 40–60 [3,5].

Fibromyalgia is a clinical syndrome characterized by a dysregulation of neuroendocrine function and/or nociceptive processing. The clinical manifestations of FM include global widespread pain often accompanied by an associated sleep disturbance [3,5,6]. There is also evidence of autonomic dysfunction suggested by associated symptoms such as irritable bowel, interstitial cystitis, temporomandibular joint dysfunction, and many others [3,4,5,6]. FM, as the most prevalent member of the CSS, poses significant diagnostic and therapeutic challenges to medicine [3,4,5,6,7]. Seventy-five percent of individuals with FM remain undiagnosed. Furthermore, from time of onset of initial symptoms, it takes on average up to 5 years for affected individuals to obtain a diagnosis [8]. This extended period between disease onset and diagnosis leads to years of unnecessary medical investigations, an exorbitant drain on valuable healthcare resources, and can result in delayed and potentially suboptimal treatment [8,9,10,11,12,13].

Musculoskeletal complaints account for over 20 million ambulatory care visits annually [3,9]. Retrospective review of emergency room records reveals that greater than 20% of these visits are due to FM. The functional and economic impact of FM is enormous, with as many as 25% of FM patients in the US receiving some form of disability or injury compensation [3,9]. Healthcare costs of FM patients are more than double that for people without FM [3,9]. Considering work absence, decreased work efficiency, healthcare costs, and litigation, overall impact of FM annually probably is in excess of $35 billion dollars. Duloy and Clemens et al., reported the high economic burden of annual medical expenses for patients with several chronic pain conditions, including FM ($4145), Rheumatoid Arthritis (RA) ($7349), and Interstitial Cystitis ($3977) (all costs adjusted to 2009 U.S. dollars) [14,15]. In fact, the negative impact on quality of life in individuals with FM is similar to those individuals afflicted with RA. Martinez et al. [16] compared a number of quality-of-life assessments in matched cohorts of FM and RA patients. There was a decrease in family income of 65% in patients with FM and 75% for subjects with RA, while 55% of patients with FM received social security aid compared to 67% with RA. Bombardier and Buchwald [17] studied 402 patients from a university-based chronic fatigue clinic. They found that individuals with comorbidity of chronic fatigue syndrome (CFS) and FM were most likely to be unemployed and use adjunctive health services. CFS and FM had similar levels of disability and healthcare utilization. Unemployment ranged from 26 to 51% and average number of visits to healthcare providers was approximately 21 visits annually [17]. Increased economic costs of FM are also noted to positively correlate with severity of disease. In a study conducted in France and Germany [18], increased costs were again associated with having FM with 75% related to a loss of work productivity. The costs increased as FM severity increased with a more than 200% difference in cost between mild and severe FM based on FM Fibromyalgia Impact Questionnaire (FIQ) scores [18].

Therapy for FM is hampered by lack of consistent, evidence-based management guidelines. Pain processing requires transmission from peripheral tissues to the brain and can be modulated by both endogenous and exogenous processes. Physical, psychological, and pharmacologic interventions can help to modulate the perception of pain in affected individuals. The initial focus and the base of any treatment protocol involves physical therapy aimed at improved conditioning. Selective serotonin reuptake inhibitors (SSRI), serotonin norepinephrine reuptake inhibitors (SNRI), alpha 2 delta ligands, and tricyclic antidepressants form the therapeutic armamentarium as we seek to modulate the neurochemical pain pathways present in the brain. Unfortunately, treatment often fails to lead to adequate recovery, frustrating both the patient and physician [3,5,6,7].

The initial 1990 American College of Rheumatology (ACR) diagnosis of FM was heavily weighted on the presence of painful tender points in a global distribution [19]. Multiple adjustments to the criteria have followed with the most recent revision centered on a combination of pain plus a variety of other symptoms [20,21]. Notwithstanding these changes, many physicians still lack the proper training to diagnose FM [22]. Patients with ill-defined symptomatology are grouped into the FM category inappropriately [22]. The most recent criteria for diagnosis are prone to error due to subjectivity in the survey elements widespread pain index (WPI) and symptom severity scale (SSS) [22,23]. In addition, a significant number of clinicians fail to use the criteria and instead utilize their “clinical acumen”, which oftentimes is incorrect [23]. In point of fact, the majority of patients who receive a diagnosis of FM from a health professional do not fulfill issued FM criteria [8,22,23].

Although many criteria have been put forth and multiple revisions have ensued over the last three decades, problems still are present with acceptance of criteria, delayed diagnoses, use of criteria in general practice, and as a result, misdiagnosis of patients remains rampant. A push to move initial identification, diagnosis, and treatment of FM to generalists rather than specialists has not enhanced diagnostics, treatment, or quality of life [24,25,26,27,28].

Discovery of a reliable biomarker for FM would be a critical step towards early intervention in this condition, as well as help to contain health care and/or legal costs. Annemans and colleagues [12] have shown that, following a diagnosis of FM, a decrease in costs generally follows, suggesting that making a diagnosis leads to savings and a decrease in resource use. The savings was observed mainly in reductions in tests and imaging, pharmaceuticals, referrals, and primary care physician visits [12]. Thus, failing to diagnose a true case of FM has its own inherent costs to society outside of the economic costs based on loss of productivity, etc. The lack of a reliable diagnostic test significantly impedes progress in understanding and treating FM. An accurate biomarker could revolutionize the diagnosis of FM, improve treatment and prognosis capabilities, and provide clues to the etiopathogenesis of the syndrome. The following review of biomarker candidates for FM encompassed use of the search engines PubMed and Google Scholar. Search terms used included Fibromyalgia, Fibrositis, Widespread Pain, central sensitivity, biomarker, metabolite, and diagnosis. The period scanned was up to November 2020.

## 3. Functional Magnetic Resonance Imaging

Functional magnetic resonance imaging (FMRI) has been utilized to document aberrant sensitization in the central nervous system (CNS) of individuals with FM [29]. In an important paper by Gracely et al., it was shown that application of moderated painful pressure in FM subjects yielded pain reports and brain activation responses that were similar to many of the effects produced by application of at least twice the pressure in control subjects. Functional activation patterns were compared in 16 FM patients and 16 matched controls. Significant increases in the FMRI signals were reflective of increases in regional cerebral blood flow. Changes in regional cerebral blood flow activity were seen in the primary and secondary sensory cortices, cerebellum, putamen, inferior parietal lobule, and superior temporal gyrus. These findings represent an experimental demonstration of the augmentation of pain processing seen in FM, which is likely mediated by multiple mechanisms and modulated by multiple factors. Drawbacks of FMRI include the fact that it is expensive, inefficient for routine clinical use, and is only available in specialized centers.

## 4. Heart Rate Variability

The stress response may elicit variable changes in heart rate, which might in turn serve as a biomarker. For example, it is possible to identify individuals within the same general group of subjects who display marked differences in magnitude and duration of their neuroendocrine responses to various stressors [30]. Heart rate variability (HRV) is accompanied by autonomic nervous system changes including rapid heart rate, orthostatic changes, Raynaud’s, and/or irritable bowel syndrome symptomatology, and therefore variability may serve as a surrogate indicator of autonomic nervous system dysfunction in some patients with FM. Studies to date have shown that FM patients electrocardiograms show significant reductions in HRV compared to normal subjects. The advantages of HRV include that it represents a noninvasive measure of sympathetic and parasympathetic balance in that it only requires electrocardiogram recordings with appropriate computer analyses. Measures such as measurements of the R-R interval, R-R interval changes between inspiration and expiration, total beats of QRS per minute, amongst others, are simple ways of assessing HRV [31]. However, overall heart rate is influenced by so many different factors, HRV becomes an unpredictable and unreliable quantitative measure for following individual subjects with FM.

## 5. Genetic Associations

Evidence of familial associations has been documented in FM with corroboration that first-degree relatives of individuals with FM patients are more likely to suffer from comorbid conditions like chronic fatigue syndrome, irritable bowel syndrome, temporomandibular joint dysfunction (TMJ), migraines, and other regional pain syndromes [32,33,34,35]. Buskila and colleagues reported on 58 offspring from 20 complete nuclear families where mothers met ACR diagnostic criteria. Of the 58 offspring, 16 (28%) were determined to have FM. Of note, there was no significant difference between offspring with FM and offspring without FM with regard to levels of anxiety, depression, global well-being, quality of life and physical functioning as assessed by validated questionnaires. Possible “candidate genes” include various serotoninergic markers including Human leukocyte antigens (HLA) and Major Histocompatibility Complex (MHC) Class I and II peptides (A, B, and DRB1), serotonin transporter promoter region (5-HTTLPR) polymorphism, T102C polymorphism of the 5-HT2A receptor gene, serotonin receptors HTR3A and HTR3B, COMT gene polymorphism, dopaminergic markers, specifically the dopamine D4 exon III 7-repeat polymorphism and substance P-related polymorphism. Other genes that have been associated with pain include single-nucleotide polymorphisms (SNP) in genes that code for melanocortin-1 receptor and neuronal cytochrome P4502D6, which are both associated with alterations in opioid analgesia. The 5-HTTLPR polymorphism has been associated with anxiety-related personality traits and stress-related depression. The COMT polymorphism has most frequently been linked to temporomandibular dysfunction, another of the CSS. A single-SNP val^158^met has been shown to play a role in variation to temporal summation to pain, whereas other SNPs exert influence on resting nociceptive sensitivity. Interestingly, certain haplotypes of the TRPV_2_ gene have been implicated as playing a protective role against the clinical symptoms of FM. In summary, a number of genetic markers and polymorphisms have been identified as showing associations in subsets of FM patients; however, none have clearly been able to show consistent variation with disease activity, nor have any been shown to display widespread clinical utility [36,37,38,39,40].

Much work is also being done on the genetic front using technology gleaned from genome-wide association studies [40]. GCH1 gene encodes the enzyme GTP cyclohydrolase 1. This enzyme is the rate-limiting enzyme in the synthesis of tetrahydrobiopterin (BH4) [41]. BH4 is an essential cofactor for nitric oxide (NO) production, and increased NO levels enhance nerve pain sensitivity. Due to this association, Kim et al. evaluated whether there was an association of the presence of this GCH1 gene polymorphism in FM. Indeed, they showed that the CCTA haplotype of the GCH1 gene was associated with protection against FM susceptibility, occurred less frequently than the CCTT haplotype, and lowered the pain sensitivity of FM patients [42]. Thus, NO may be involved in modulating pain perception in FM patients, and certain GCH1 haplotypes may be protective against susceptibility and pain sensitivity in FM. It should be noted that this study, although done in a large cohort of 409 patients with FM and 422 controls, was conducted in an entirely Korean population. It has not been determined what the prevalence of this polymorphism is in other FM cohorts.

## 6. IL-8 and MCP-1

Another set of putative biomarkers includes IL-8 and MCP-1 [43]. Ang and colleagues studied 28 subjects with FM. They correlated changes from week 1 through week 12 between recorded values on the brief pain inventory (BPI) and plasma concentrations of IL-8 and MCP-1. For each unit increase in the change of BPI pain severity, IL-8 increased by 2.5 pg/mL (*p* = 0.03) and MCP-1 increased by 9.4 pg/mL (*p* = 0.006). Body mass index (BMI), medications, severity of depression, and overall FM burden were not found to significantly be associated with either IL-8 or MCP-1. They theorized that there are increased amounts of skin mast cells in FM [44,45] along with the already known elevated levels of substance P and CRH, both elevated in the CSF of FM patients [46,47,48,49], and these compounds activate the mast cells in the skin and lead to release of the compounds IL-8 and MCP-1. Although initially intriguing, these results have not been reproduced in a large scale study nor lent themselves towards targeted approaches to treat FM.

## 7. Obesity

Obesity has been explored as a possible biomarker [50]. Okifuji and colleagues evaluated 38 FM subjects specifically looking at associations with obesity. In their study, Body Mass Index (BMI) was the primary indicator of obesity. They found that approximately 50% of the subjects were obese and an additional 21% were overweight. Positive correlations were found between BMI and IL-6 levels (r = 0.52) and epinephrine (r = 0.54), with weaker associations with cortisol and C-reactive protein (CRP) (0.32 and 0.37), respectively. BMI was weakly associated with disturbed sleep; total sleep time (r = −0.56) and sleep efficiency (r = −0.44). No associations between the Fibromyalgia Impact Questionnaire (FIQ) and BMI were found. Two areas of caution in the interpretation of these results include the fact that obesity prevalence in this study was 50% compared to 30% in the general population and the potential effects of aberrant hypothalamic-pituitary-adrenal (HPA) regulation coupled with obesity and FM.

## 8. Tender Point Counts

Global widespread pain has been one of the prime characteristics of identifying individuals with FM. Earlier versions of diagnostic criteria included “tender point counts” as crucial in identifying if individuals had FM or not. Earlier randomized prospective studies showed improvement in clinical pain correlating with decreases in tender point count; however, other studies have failed to show these same findings [51]. In a large analysis of 627 outpatients with FM, the rheumatology distress index was linearly correlated to the tender point count (r^2^ = 0.30). There was no specific abnormality of FM associated with very high levels of tender points other than the level of distress. The suggestion was that tender points might function as a form of “erythrocyte sedimentation rate” for patients with FM. As a result, the diagnostic significance of tender points was thrown into question, and they were no longer included as essential in more recent versions of diagnostic criteria, but even that move has been subject to debate [52].

## 9. Pressure Pain Thresholds

Alternative evoked pain measures (other than tender point counts and dolorimetry) have been sought for a number of years. This is because these latter two approaches can both be modulated by levels of distress and/or expectancy. Pressure pain thresholds, and heat and cold pain thresholds, have been utilized in research settings and are not yet automated to the point of being useful as clinical/bedside parameters for clinical assessment [53,54,55,56,57]. Despite that, a number of studies have consistently found that pain and thermal thresholds are altered in the setting of FM. Furthermore, pressure pain testing that applies stimuli randomly may be advantageous for use over traditional tender point counts or dolorimetry.

## 10. Sleep Architecture

Sleep architecture has been shown to be disrupted with the presence of alpha wave intrusion on polysomnography graphs since the classic experiments of Moldofsky [58]. The original published group involved 10 individuals; ages 37–64 with FM and 6 controls, ages 19–24. All individuals underwent Stage 4 sleep deprivation with alpha rhythms in non-rapid eye movement (NREM) sleep observed (also called alpha-delta sleep). In the control group, Stage 4 deprivation led to temporary myalgias and mood disturbances similar to symptoms seen in FM group. Thus, the suggestion that a non-restorative sleep pattern was inherent in FM received experimental confirmation. Although this finding is well established, it is not specific for FM [58]. Actigraphs are devices which assess movement during sleep and can help in painting a general picture of sleep disruption for affected individuals. Many disorders such as Restless Legs Syndrome (RLS), amongst others, lead to actigraph abnormalities. RLS is one of many CSS that are found to be frequently comorbid with FM [59]. As a result, this type of methodology cannot serve as a specific biomarker for FM subjects [60].

## 11. Hypothalamic Pituitary Adrenal Axis

The most consistent finding regarding the hypothalamic-pituitary–adrenal axis system in FM subjects has been a flattening of the diurnal plasma cortisol level with an elevated trough [61]. Crofford and colleagues studied 40 patients with either FM (*n* = 13), FM and chronic fatigue syndrome (CFS) (*n* = 12), or CFS (*n* = 15) who were matched by age (18–65), sex, and menstrual status to healthy controls. Blood collection was done every 10 min over 24 h for analysis of adrenocorticotropic hormone (ACTH) and cortisol. Samples were evaluable for ACTH in 36 subject pairs and for cortisol in 37 subject pairs. There was a significant delay in the rate of decline from acrophase to nadir for cortisol levels in patients with FM (*p* < 0.01). However, inconsistent results have been obtained in multiple studies of basal plasma cortisol, salivary basal and diurnal cortisol, and urinary cortisol, making these measures generally unreliable. Therefore, these measurements cannot be used on a widespread basis.

## 12. Anti-Serotonin Antibodies

The presence of anti-serotonin antibodies was reported by Klein and colleagues in 1992 [62]. In order to evaluate whether patients with FM suffered from an “autoimmune disorder”, they tested sera from 50 FM patients by enzyme-linked immunosorbent assay and immunofluorescence test; 74% of those tested had antibodies against serotonin and gangliosides. Anti-serotonin antibodies were not found in rheumatoid arthritis (RA), polymyalgia rheumatica or other collagen disorders [62]. The authors speculated that these findings may convey some relevance due to their absence in the other rheumatic disorders that were tested. In a much larger study of 203 patients with FM and 64 pain-free controls, Werle and colleagues found that, although there was an elevated prevalence of antibodies against serotonin in patients with FM, the measurement of these antibodies had no diagnostic relevance [63]. Two other studies conducted by Russell and Bennett also did not support an association of these antibodies with FM [64,65].

## 13. Substance P, Nerve Growth Factor, Brain-Derived Neurotrophic Factor, Glutamate, Tryptophan, Melatonin

Neuropeptide levels (Substance P [45,46], Nerve Growth Factor (NGF) [66,67], Brain-Derived Neurotrophic Factor (BDNF) [68], low tryptophan (TRP) [69] have all been documented reproducibly in FM subjects relative to normal controls. In a classic study, Russell measured cerebrospinal fluid (CSF) substance P (SP) levels via radioimmunoassay. In 32 FM patients and 30 normal control subjects, CSF SP levels were 3-fold higher in FM patients than normal controls (*p* < 0.001). This finding replicated prior studies of Vaeroy [48]. Giovengo showed elevated levels of NGF in the CSF through the use of enzyme immunoassay. NGF measured in patients with FM was increased (41.8 +/− 12.7 pg/mL) compared to controls (9.1 +/− 4.1 pg/mL). Notably, concentrations of NGF in conditions with secondary FM were not elevated compared to controls. The authors concluded that the increased concentrations of NGF in patients with FM might suggest a central mechanism. BDNF is important in synaptic plasticity and neuronal survival in the central and peripheral nervous systems (CNS and PNS). As such, it would naturally be possibly implicated as having a role in central pain mechanisms. BDNF serum concentrations in 41 FM patients were compared to 45 controls. Serum levels of BDNF in FM patients (19.6 ng/mL; SD 3.1) were increased as compared to controls (16.8 ng/mL; SD 2.7; *p* < 0.0001). Of note, plasma samples of NGF and BDNF have not displayed the elevations in FM subjects relative to non-pain controls seen in CSF or serum in multiple studies [70,71]. Sarchielli looked at CSF levels of BDNF in 20 chronic migraine (CM), 20 FM, and 20 control subjects. There was a significant positive correlation between CSF values of BDNF and NGF (r = 0.61, *p* < 0.001; r = 0.53, *p* < 0.01) and glutamate (r = 0.44, *p* < 0.02; r = 0.51, *p* < 0.91 (in CM and FM patients, respectively.

Tryptophan metabolism has been implicated in FM pathology due to possible roles of its metabolites in sleep, fatigue, and pain propagation. Changes in melatonin (MT) secretion and/or availability may have consequences on sleep preservation. Two MT precursors (tryptophan and serotonin) which affect both sleep and pain perception appear to be low in patients with FM. Previous studies have tried to determine whether serum MT (s-MT) level was also low in these patients. Eight patients with FM and 8 controls were included in the study. FM patients had a 31% lower MT secretion than controls during the hours of darkness. Also, the s-MT peak value was significantly lower in FM [72]. Plasma tryptophan and transport ratios of tryptophan have both been shown to be significantly lower in FM relative to controls. In a study of 29 patients with FM and 30 non-pain controls, transport ratios of tryptophan were shown to be significantly decreased (*p* < 0.01) relative to the control group. Findings suggested that lower brain serotonin levels might be associated with decreased transport ratios of plasma tryptophan [69].

## 14. Skeletal Muscle Abnormalities

Various muscle abnormalities have been reported in FM [73]. These abnormalities are histologically distinct and include so-called ragged red fibers with abnormal mitochondria, rubber band morphology: (primarily described in the quadriceps) [74], decreased capillary numbers [75,76], thickening of capillary endothelium [73], abnormal mitochondria [77], and sarcolemma membrane damage [78]. None of these findings are specific. For example, ragged red fibers with abnormal mitochondria is a characteristic finding of other conditions; most frequently mitochondrial myopathy. Some of these histologic abnormalities have been associated with clinical symptoms; for example, decreased capillary numbers have been associated with pain, thickened capillary endothelium is associated with fatigue and abnormal mitochondria is associated with weakness. All of these histologic findings lack the reproducibility and or standardized methodology needed to verify the findings [73]. Furthermore, muscle biopsy is invasive with significant post-procedure healing and pain, making widespread use of these techniques unlikely.

## 15. Small-Fiber Neuropathy

One of the more recently identified abnormalities that has been consistent is indicative of a small-fiber neuropathy (SFN) [79]. Several groups including our own have found evidence for epidermal nerve fiber dropout. We reported greater than 60 percent of our subjects with FM with abnormal skin biopsy findings as well as evidence for probable small-fiber neuropathy [79,80,81]. We studied a cohort of 61 patients, of which 34 (61%) were found to have reduced intraepidermal nerve fiber density (IENFD) by skin punch biopsy, which indicated evidence for a small-fiber neuropathy [80]. Of note, 24 of 34 (71%) patients had laboratory evidence that revealed an underlying etiology for the SFN. These previously undiagnosed causes of SFN were glucose dysmetabolism, Sjogren’s syndrome, elevated ESR and ANA, vitamin B6 or B12 deficiency, and Fabry’s disease. It is illuminating that laboratories surveilling for these types of conditions are not amongst the typical ones obtained during normal evaluation of FM patients. This is part of the growing evidence that suggests that the presence of SFN is associated with more severe FM. Perhaps lack of elucidating a proper underlying etiology leads to therapy which is being directed to an inappropriate primary source. Oftentimes in patients that are found to have SFN from other causes, their symptoms will precede a positive skin biopsy and careful history intake might reveal the same in FM. Therapy directed at neuroinflammatory or neuroimmune mediators may provide more directed approaches for this subset of individuals. In any event, these findings attest to the diverse etiologies leading towards this clinical phenotype. Although skin biopsy is invasive, in difficult-to-treat FM, which is resistant to standard treatments, it may be entirely justifiable.

## 16. Mononuclear Cell Cytokine Assays

Wallace and colleagues have reported on the development of a chemokine and cytokine multiplex immunoassay after mitogenic stimulation with the goal of improving and achieving an accurate diagnosis of FM. Their study involved the use of isolated mononuclear cell. These monocytes were stimulated and supernatants were assayed for measurement of the following cytokines and chemokines of interest; IL-6, IL-8, MIP-1 alpha and MIP-1 beta. The analyses included 160 patients with FM, 98 with RA, and 100 with SLE who were subsequently compared to 119 controls. Sensitivity, specificity, positive predictive, and negative predictive value for having FM compared to controls (SLE and RA) were 93, 89, 92, and 91%, respectively. The authors concluded that evaluating cytokine and chemokine profiles in stimulated cells revealed patterns that are uniquely present in patients with FM. They felt that this assay could be a useful tool in differentiating systemic inflammatory autoimmune processes from FM and healthy controls [82].

## 17. Metabolomics and Metabolic Fingerprinting

Hackshaw and colleagues identified a biomarker “fingerprint” for FM in serum using an infrared microspectroscopy-chemometric (IRMS-C) approach [83]. IRMS-C utilizes a laser which causes vibrations in characteristic patterns after interaction with functional groups (e.g., methyl, carbonyl, etc.) in biological samples of interest for rapid, high-throughput, non-destructive analysis of a wide range of sample types [84,85]. This technique allows for rapid analysis of a wide range of sample types with minimal sample preparation and very small volumes. Operators require minimal training and analyses are relatively low cost. IRMS-C is being increasingly used in a wide range of medical areas. [84,86,87]. Additional details of the methodology are found in prior publications [83,87,88]. Hackshaw and colleagues were the first group to utilize this vibrational spectroscopy technology in FM. FM patients were clearly distinguished from RA and OA groups by pattern recognition software with 100% accuracy and no misclassifications [83]. Metabolomics analyses suggested that changes in tryptophan catabolism might have been the reasons for the spectral differentiation. Malatji [89] used NMR metabolomics on urine to identify a diagnostic biomarker profile in FM. Succinic acid, taurine, creatine correlated with pain and fatigue symptoms and hippuric, 2-OH-isobutyric and lactic acid appeared to suggest activation related to the gut microbiome. More recently, Hackshaw and colleagues utilized vibrational spectroscopy to differentiate patients with FM from those with rheumatoid arthritis (RA), osteoarthritis (OA), and systemic lupus erythematosus (SLE) [90]. Whole blood samples from FM (*n* = 50), RA (*n* = 29), OA (*n* = 19), or SLE (*n* = 23) were analyzed [90]. Portable FT-IR and FT-Raman microscope were used to obtain spectra followed by metabolomics analysis via ultra-HPLC (uHPLC), coupled to tandem MS/MS. IR and Raman spectral signatures were identified by pattern recognition analysis and found to be distinct. FM, RA, and SLE clustered together with no misclassifications (*p* < 0.05, and interclass distances > 2.5). Spectra correlated (r = 0.95 and 0.83 for IR and Raman, respectively) with FM pain severity measured with fibromyalgia impact questionnaire (FIQR) assessments. Protein backbones and pyridine-carboxylic acids appeared to drive the discrimination ability. Further analysis of unique metabolites driving discrimination was achieved by uHPLC-PDA-MS/MS. These studies may lead to specific therapeutic targets for establishing serologic biomarkers of FM-associated pain. The approach of the Hackshaw group has been independently corroborated by Passos, who has also utilized vibrational spectroscopy. Passos and colleagues evaluated 126 controls and 126 patients with FM using Fourier Transform Infrared (FTIR) spectroscopy in conjunction with chemometric techniques. A genetic algorithm with linear discriminant analysis was found which achieved their best diagnostic results with a sensitivity of 89.5% in an external test set. Amide II (1545 cm) and proteins 1425 cm were identified to be discriminant features. Their results reinforce the prior results of Hackshaw in showing the potential of vibrational spectroscopy with multivariate analysis as a tool to screen and detect patients with FM in a rapid, low-cost, and minimally invasive manner [91].

Vibrational (mid-IR and Raman) spectroscopy is a way of providing a non-destructive biochemical fingerprint or signature on a wide range of biologic samples. In contrast, NMR spectroscopy and mass spectrometry (MS) techniques, although providing potential for specificity of the biologic sample of interest, require costly instrumentation, are labor-intensive, and involve complex sample preparation. Thus, this type of methodology is not point-of-care ready. Advances in the last few years through micro-electro-mechanical systems (MEMS) technological developments has led to miniaturization of vibrational spectrometers and the potential for bedside clinical analyses in the future through the use of portable units [92]. Interestingly, Hackshaw noted that the intensity of the spectral bands were associated with FM disease activity [83,90], suggesting that vibrational spectroscopy may be useful to monitor the state of disease activity in these patients. The importance of these findings cannot be understated, because changes in spectrum during different disease states may correlate with different metabolic signatures. These alternate signatures may portend different therapeutic approaches or may suggest new therapeutic targets.

## 18. Mu Opioid Positive B Lymphocytes

Recently, the mu opioid receptor on B lymphocytes has been evaluated as a potential biomarker for FM and osteoarthritis (OA) patients [93]. Blood samples were collected from three groups of female patients with FM, OA, or pain-free controls. Immunophenotyping analysis showed that percentages of Mu-positive B cells were statistically lower in FM and OA patients than in pain-free subjects. FM and OA subjects were all categorized in terms of mild, moderate, and severe pain intensity categories. FM with moderate and severe levels of pain showed significantly lower percentages of Mu+ B cells than negative control group patients (*p* < 0.001). OA moderate and severe pain patients showed significantly lower Mu+ B cell percentages than mild pain OA and negative control patients (*p* < 0.01). Possibilities posed by these studies include the use of immunophenotyping as a biomarker for chronic pain and ease of this type of peripheral analysis.

## 19. Summary

In summary, there are many promising research fronts that are being pursued for identification of a biomarker in FM. As these investigations proceed, we continue to learn more about the pathogenesis of this condition. Most probably, FM is a clinical phenotypic end point approached through diverse pathogenetic mechanisms. As a result, it is probable that multiple biomarkers may need to be utilized depending upon the clinical presentation. Whether the diversity of the inciting mechanisms might be reflected in subsequently identified biomarker(s) awaits future discovery. If found, they would help provide clues towards the most appropriate treatment avenues.

Our current approach towards treatment of FM involves physical, psychological, and pharmacologic interventions, all aimed at modulating the perception of pain and pain propagation in affected individuals. Physical therapy is the initial focus characterized by a self-directed or interventional teaching program followed by a self-directed exercise approach. The aim of such programs is to improve conditioning. Later therapeutic options include attempts to modulate the neurochemical pain pathways present in the brain. Tricyclic antidepressants, selective serotonin reuptake inhibitors (SSRI), serotonin norepinephrine reuptake inhibitors (SNRI), and alpha 2 delta ligands are the mainstays of the therapeutics used for these purposes. Treatment is often inadequate, resulting in incomplete recovery or pain improvement levels of only 30% over baseline, which limits the potential efficacy of these therapeutics [28]. The list of putative biomarkers is summarized in Table 1. Perhaps, going forward, we may need to be resigned to the fact that multiple biomarkers may be the most appropriate approach for diagnosis of a condition as complex as FM. As an example, it is likely that individuals with FM and an associated small-fiber neuropathy might have arrived at that clinical end point via underlying mechanisms of neuro-immune inflammation. Further clinical investigations targeting potential etiologies for abnormal IENFD leading to small-fiber neuropathy might point towards alternative and more directed treatment approaches. Appropriate therapy for a portion of these patients might include treatments targeting neuro-immune modulation (corticosteroids, intravenous gammaglobulin, etc. [76]). These types of agents are inappropriate for the vast majority of non-small-fiber neuropathy patients. Targeted putative biomarkers such as skin biopsy would be able to identify these groups and more appropriately direct treatment. Other individuals who present with symptoms of myalgia and profound weakness might benefit from muscle biopsy. A diagnosis of “ragged muscle fibers” might point towards a mitochondrial myopathy mimicking as “Fibromyalgia”. Therefore, it appears that the future for FM may include selective use of multiple biomarkers. Our use of a particular marker would be directed by clinical presentation and therapeutic responsiveness to typical medication regimens. Whether newer technologies such as vibrational spectroscopy or other modalities would have the capability of more globally identifying FM amidst the morass of potential etiologies awaits further research. Therefore, as discovery continues for candidate biomarkers, we can hope that subsequent discoveries also point us towards the most appropriate therapeutic options for our patients.

## Figures and Tables

**Table 1:** Listing of putative biomarkers and corresponding references.

Potential Biomarkers for Fibromyalgia	Reference
Functional Magnetic Resonance Imaging (FMRI)	[29]
Body Mass Index (BMI)	[50]
Heart Rate Variability	[30,31]
Genetic Associations:	[32,33,34,35]
Serotoninergic Markers:	
HLA A, B, DRB1	[36]
Polymorphisms:	
5HTTLPR	
T102C of HT2A,	
HTR3A	[37]
HTR3B	
COMT	[38]
Dopamine D4 Exon III 7-repeat	[39]
GCH1 Polymorphism	[41,42]
Muscle Abnormalities:	[73]
Rubber Band Morphology (Quadriceps)	[74]
Decreased Capillary numbers	[75,76]
Thickening of capillary endothelium	[73]
Abnormal mitochondria	[77]
Sarcolemmal damage	[78]
Neuropeptides:	
Substance P (CSF)	[48,49]
Nerve Growth Factor (CSF)	[66,67]
Brain-Derived Neurotrophic Factor (BDNF) (serum)	[68]
Tryptophan (plasma)	[69]
NGF (plasma)	[70,71]
BDNF (plasma)	[70,71]
IL8 and MCP-1	[43]
Tender Point Counts	[51,52]
Mast Cells (Skin)	[44,45]
Corticotropin Releasing Factor (CSF)	[46,47]
Melatonin	[72]
ACTH and Cortisol	[47]
Cytokine Multiplex Assay	[82]
Pressure Point Thresholds	[53,54,55]
Heat and Cold Pain Thresholds	[56,57]
Sleep Architecture Disruption	[58,59,60]
Cortisol Levels/Hyp-Pit-Adr Axis	[61]
Anti-Serotonin Antibodies	[62,63,64,65]
Vibrational Spectroscopy Fingerprinting	[83,90]
NMR Metabolomics	[89]
Mu Opioid Receptor Positive B lymphocytes	[40,41,42]

## Data Availability

Not applicable.

## References

[1] Biomarkers Definitions Working Group (2001). Biomarkers and surrogate endpoints: Preferred definitions and conceptual framework. Clin. Pharmacol. Ther..

[2] Koulman A., Lane G.A., Harrison S.J., Volmer D.A. (2009). From differentiating metabolites to biomarkers. Anal. Bioanal. Chem..

[3] Smith H.S., Harris R., Clauw D. (2011). Fibromyalgia: An afferent processing disorder leading to a complex pain generalized syndrome. Pain Physician.

[4] Yunus M.B. (2008). Central sensitivity syndromes: A new paradigm and group nosology for fibromyalgia and overlapping conditions, and the related issue of disease versus illness. Semin. Arthritis Rheum..

[5] Clauw D.J., Fibromyalgia A. (2014). Clinical review. JAMA.

[6] Clauw D.J., Crofford L.J. (2003). Chronic widespread pain and fibromyalgia: What we know, and what we need to know. Best Pract. Res. Clin. Rheumatol..

[7] Clauw D.J. (2009). Fibromyalgia: An overview. Am. J. Med..

[8] Wallit B., Katz R.S., Bergman M.J., Wolfe F. (2016). Three quarters of persons in the US population reporting a clinical diagnosis of fibromyalgia do not satisfy fibromyalgia criteria: The 2012 national health interview survey. PLoS ONE.

[9] Wolfe F. (1996). The fibromyalgia syndrome: A consensus report on fibromyalgia and disability. J. Rheumatol..

[10] White L.A., Birnbaum H.G., Kaltenboeck A., Tang J., Mallett D., Robinson R.L. (2008). Employees with fibromyalgia: Medical comorbidity, healthcare costs, and work loss. J. Occup. Environ. Med..

[11] Silverman S., Dukes E.M., Johnston S.S., Brandenburg N.A., Sadosky A., Huse D.M. (2009). The economic burden of fibromyalgia: Comparative analysis with rheumatoid arthritis. Curr. Med. Res. Opin..

[12] Annemans L., Wessely S., Spaepen E., Caekelbergh K., Caubere J.P., Lay K.L., Taieb C. (2008). Health economic consequences related to the diagnosis of fibromyalgia syndrome. Arthritis Rheum..

[13] Chandran A., Schaefer C., Ryan K., Baik R., Mc Nett M., Zlateva G. (2012). The comparative economic burden of mild, moderate, and severe fibromyalgia: Results from a retrospective chart review and cross-sectional survey of working-age US adults. J. Manag. Care Pharm..

[14] Duloy A.M.S., Calhoun E.A., Clemens J.Q. (2007). Economic impact of chronic prostatitis. Curr. Urol. Rep..

[15] Clemens J.Q., Markossian T., Calhoun E.A. (2009). Comparison of the economic impact of chronic prostatitis/chronic pelvic pain syndrome and interstitial cystitis/painful bladder syndrome. Urology.

[16] Martinez J.E., Ferraz M.B., Sato E.I., Atra E. (1995). Fibromyalgia versus rheumatoid arthritis: A longitudinal comparison of the quality of life. J. Rheumatol..

[17] Bombardier C., Buchwald D. (1996). Chronic fatigue, chronic fatigue syndrome, and fibromyalgia: Disability and health-care use. Med. Care.

[18] Winkelmann A., Perrot S., Schaefer C., Ryan K., Chandran A., Sadosky A., Zlatev G. (2011). Impact of fibromyalgia severity on health economic costs. Appl. Health Econ. Health Policy.

[19] Wolfe F., Smyth H.A., Yunus M.B., Bennett R.M., Bombardier C., Goldenberg D.L., Tugwell P., Campbell S.M., Abeles M., Clark P. (1990). The American college of rheumatology 1990 criteria for the classification of fibromyalgia. Arthritis Rheum..

[20] Wolfe F., Clauw D.J., Fitzcharles M.A., Goldenberg D.L., Katz R.S., Mease P., Russell A.S., Russell I.J., Winfield J.B., Yunus M.B. (2010). The American college of rheumatology preliminary diagnostic criteria for fibromyalgia and measurement of symptom severity. Arthritis Care Res..

[21] Ablin J.N., Wolfe F.A. (2017). Comparative evaluation of the 2011 and 2016 criteria for fibromyalgia. J. Rheum..

[22] Gittins R., Howard M., Ghodke A., Ives T.J., Chelminski P. (2018). The accuracy of a fibromyalgia diagnosis in general practice. Pain Med..

[23] Kumbhare D., Ahmed S., Sander T., Grosman-Rimon L., Srbely J. (2018). A Survey of physicians’ knowledge and adherence to the diagnostic criteria for fibromyalgia. Pain Med..

[24] Eich W., Hauser W., Arnold B., Jäckel W., Offenbächer M., Petzke F., Schiltenwolf M., Settan M., Sommer C., Tölle T. (2012). Fibromyalgia syndrome. Definition, classification, clinical diagnosis and prognosis. Schmerz.

[25] Fitzcharles M.A., Shir Y., Ablin J.N., Buskila D., Amital H., Henningsen P., Häuser W. (2013). Classification and clinical diagnosis of fibromyalgia syndrome: Recommendations of recent evidence-based interdisciplinary guidelines. Evid. Based Complement. Altern. Med..

[26] Fitzcharles M.A., Ste-Marie P.A., Goldenberg D.L., Pereira J.X., Abbey S., Choinière M., Ko G., Moulin D.E., Panopalis P., Proulx J. (2013). 2012 Canadian guidelines for the diagnosis and management of fibromyalgia syndrome: Executive summary. Pain Res. Manag..

[27] Hauser W., Bernardy K., Wang H., Kopp I. (2012). Methodological fundamentals of the development of the guideline. Schmerz.

[28] Hackshaw K. (2020). Assessing our approach to diagnosing fibromyalgia. Exp. Rev. Mol. Diag..

[29] Gracely R.H., Petzke F., Wolf J.M., Clauw D.J. (2002). Functional magnetic resonance imaging evidence of augmented pain processing in fibromyalgia. Arthritis Rheum..

[30] Negrao A.B., Deuster P.A., Gold P.W., Singh A., Chrousos G.P. (2000). Individual reactivity and physiology of the stress response. Biomed. Pharmacother..

[31] Staud R. (2008). Heart rate variability as a biomarker of fibromyalgia syndrome. Fut. Rheumat..

[32] Arnold L.M., Hudson J.I., Hess E.V., Ware A.E., Fritz D.A., Auchenbach M.B., Starck L.O., Keck P.E. (2004). Family study of fibromyalgia. Arthritis Rheum..

[33] Buskila D., Neumann I., Hazanov I., Carmi R. (1996). Familial aggregation in the fibromyalgia syndrome. Semin. Arthritis Rheum..

[34] Kato K., Sullivan P.F., Evengard B., Pedersen N.L. (2006). Chronic widespread pain and its comorbidities: A population-based study. Arch. Intern. Med..

[35] Kato K., Sullivan P.F., Evengard B., Pedersen N.L. (2008). A population-based twin study of functional somatic syndromes. Psychol. Med..

[36] Mogil J.S., Yu L., Basbaum A.I. (2000). Pain genes? Natural variation and transgenic mutants. Ann. Rev. Neurosci..

[37] Diatchenko L., Slade G.D., Nackley A.G., Bhalang K., Sigurdsson A., Belfer I., Goldman D., Xu K., Shabalina S.A., Shabalina S.A. (2005). Genetic basis for individual variations in pain perception and the development of a chronic pain condition. Hum. Mol. Genet..

[38] Diatchenko I., Nackley A.G., Slade G.D., Bhalang K., Belfer I., Max M.B., Goldman D., Maixner W. (2006). Catechol-O-methyltransferase gene polymorphisms are associated with multiple pain evoking stimuli. Pain.

[39] Buskila D., Cohen H., Neumann L., Ebstein R.P. (2004). An association between fibromyalgia and the dopamine D4 receptor exon III repeat polymorphism and relationship to novelty seeking personality traits. Mol. Psychiatry.

[40] Park D.J., Lee S.S. (2017). New insights into the genetics of fibromyalgia. Korean J. Intern. Med..

[41] Thony B., Auerbach G., Blau N. (2000). Tetrahydrobiopterin biosynthesis, regeneration and functions. Biochem. J..

[42] Kim S.K., Kim S.H., Nah S.S., Lee J.H., Hong S.J., Kim H.S., Lee H.S., Kim H.A., Joung C.I., Bae J. (2013). Association of guanosine triphosphate cyclohydrolase 1 gene polymorphisms with fibromyalgia syndrome in a Korean population. J. Rheumatol..

[43] Ang D.C., Moore M.N., Hilligoss J., Tabbey R. (2011). MCP-1 and IL-8 as pain biomarkers in fibromyalgia: A pilot study. Pain Med..

[44] Blanco I., Beritze N., Arguelles M., Cárcaba V., Fernández F., Janciauskiene S., Oikonomopoulou K., De Serres F.J., Fernández-Bustillo E., Hollenberg M.D. (2010). Abnormal overexpression of mastocytes in skin biopsies of fibromyalgia patients. Clin. Rheumatol..

[45] Enestrom S., Bengtsson A., Frodin T. (1997). Dermal IgG deposits and increase of mast cells in patients with fibromyalgia—relevant findings or epiphenomena?. Scand. J. Rheumatol..

[46] Mclean S.A., Williams D.A., Stein P.K., Harris R.E., Lyden A.K., Whalen G., Park K.M., Sen A., Liberzon I., Gracely R.H. (2006). Cerebrospinal fluid corticotropin-releasing factor concentration is associated with pain but not fatigue symptoms in patients with fibromyalgia. Neuropsychopharmacology.

[47] Riedel W., Schlapp U., Leck S., Netter P., Neeck G. (2002). Blunted ACTH and cortisol responses to systemic injection of corticotropin-releasing hormone (CRH) in fibromyalgia: Role of somatostatin and CRH-binding protein. Ann. N. Y. Acad. Sci..

[48] Vaeroy H., Helle R., Forre O., Kass E., Terenius L. (1988). Elevated CSF levels of substance P and high incidence of Raynaud phenomenon in patients with fibromyalgia: New features for diagnosis. Pain.

[49] Russell I.J., Orr M.D., Littman B., Vipraio G.A., Alboukrek D., Michalek J.E., Lopez Y., Mackillip F. (1994). Elevated cerebrospinal fluid levels of substance P in patients with the fibromyalgia syndrome. Arthritis Rheum..

[50] Okifuji A., Bradshaw D.H., Olson C. (2009). Evaluating obesity in fibromyalgia: Neuroendocrine biomarkers, symptoms and functions. Clin. Rheum..

[51] Arnold L.M., Hess E.V., Hudson J.I., Welge J.A., Berno S.E., Keck P.E. (2002). A randomized placebo-controlled, double-blind, flexible -dose study of fluoxetine in the treatment of women with fibromyalgia. Am. J. Med..

[52] Wolfe F. (1997). The relation between tender points and fibromyalgia symptom variables: Evidence that fibromyalgia is not a discrete disorder in the clinic. Ann. Rheum. Dis..

[53] Petzke F., Gracely R.H., Park K.M., Ambrose K., Clauw D.J. (2003). What do tender points measure? Influence of distress on 4 measures of tenderness. J. Rheumatol..

[54] Harris R.E., Gracely R.H., McLean S.A., Williams D.A., Giesecke T., Petzke F., Sen A., Clauw D.J. (2006). Comparison of clinical and evoked pain measures in fibromyalgia. J. Pain.

[55] Petzke F., Clauw D.J., Ambrose K., Gracely R.H. (2003). Increased pain sensitivity in fibromyalgia: Effects of stimulus type and mode of presentation. Pain.

[56] Kosek E., Hansson P. (1997). Modulatory influence on somatosensory perception from vibration and heterotopic noxious conditioning stimulation (HNCS) in fibromyalgia patients and healthy subjects. Pain.

[57] Arroyo J.F., Cohen M.L. (1993). Abnormal responses to electrocutaneous stimulation in fibromyalgia. J. Rheumatol..

[58] Moldofsky H., Scarisbrick P., England R., Smythe H. (1975). Musculoskeletal symptoms and non-REM sleep disturbance in patients with “fibrositis syndrome” and healthy subjects. Psychosom. Med..

[59] Branco J., Atalaia A., Paiva T. (1994). Sleep cycles and alpha-delta sleep in fibromyalgia syndrome. J. Rheumatol..

[60] Edinger J.D., Wohlgemuth W.K., Krystal A.D., Rice J.R. (2005). Behavioral insomnia therapy for fibromyalgia patients: A randomized clinical trial. Arch. Int. Med..

[61] Crofford L.J., Young E.A., Engleberg N.C., Korszun A., Brucksch C.B., McClure L.A., Brown M.B., Demitrack M.A. (2004). Basal circadian and pulsatile ACTH and cortisol secretion in patients with fibromyalgia and/or chronic fatigue syndrome. Brain Behav. Immun..

[62] Klein R., Bänsch M., Berg P.A. (1992). Clinical relevance of antibodies against serotonin and gangliosides in patients with primary fibromyalgia syndrome. Psychoneuroendocrinology.

[63] Werle E., Fischer H.P., Müller A., Fiehn W., Eich W. (2001). Antibodies against serotonin have no diagnostic relevance in patients with fibromyalgia syndrome. J. Rheumatol..

[64] Russell I.J. (1998). Advances in fibromyalgia: Possible role for central neurochemicals. Am. J. Med. Sci..

[65] Vedderci I., Bennett R.M. (1995). An analysis of antibodies to serotonin receptors in fibromyalgia. J. Musculoskelet. Pain.

[66] Giovengo S.L., Russell I.J., Larson A.A. (1999). Increased concentrations of nerve growth factor in cerebrospinal fluid of patients with fibromyalgia. J. Rheum..

[67] Sarchielli P., Mancini M.L., Floridi A., Coppola F., Rossi C., Nardi K., Acciarresi M., Alberto Pini L., Calabresi P. (2007). Increased levels of neurotrophins are not specific for chronic migraine: Evidence from primary fibromyalgia syndrome. J. Pain.

[68] Laske C., Stransky E., Eschweiler G.W., Klein R., Wittorf A., Leyhe T., Richartz E., Köhler N., Bartels M., Buchkremer G. (2007). Increased BDNF serum concentration in fibromyalgia with or without depression or antidepressants. J. Psyc. Res..

[69] Yunus M.B., Dailey J.W., Aldag J.C., Masi A.T., Jobe P.C. (1992). Plasma tryptophan and other amino acids in primary fibromyalgia: A controlled study. J. Rheumatol..

[70] Baumeister D., Eich W., Saft S., Geisel O., Hellweg R., Finn A., Svensson C.I., Tesarz J. (2019). No evidence for altered plasma NGF and BDNF levels in fibromyalgia patients. Sci. Rep..

[71] Jablochkova A., Bäckryd E., Kosek E., Mannerkorpi K., Ernberg M., Gerdle B., Ghafouri B. (2019). Unaltered low nerve growth factor and high brain-derived neurotrophic factor levels in plasma from patients with fibromyalgia after a 15-week progressive resistance exercise. J. Rehab. Med..

[72] Wikner J., Hirsch U., Wetterberg L., Rojdmark S. (1998). Fibromyalgia—A syndrome associated with decreased nocturnal melatonin secretion. Clin. Endocrinol..

[73] Olsen N.H., Park J.H. (1998). Skeletal muscle abnormalities in patients with fibromyalgia. Amer. J. Med. Sci..

[74] Bartels E.M., Danneskiold-Samsoe B. (1986). Histological abnormalities in muscle from patients with certain types of fibrositis. Lancet.

[75] Drewes A.M., Andreasen A., Schroder H.D., Hogsaa B., Jennum P. (1993). Pathology of skeletal muscle in fibromyalgia: A histoimmunochemical and ultrastructural study. Br. J. Rheumatol..

[76] Bengtsson A., Henriksson K.G., Larsson J. (1986). Reduced high-energy phosphate levels in the painful muscles of patients with primary fibromyalgia. Arthritis Rheum..

[77] Lindh M.H., Johansson L.G., Hedberg M., Henning G.B., Grimby G. (1995). Muscle fiber characteristics, capillaries and enzymes in patients with fibromyalgia and controls. Scand. J. Rheumatol..

[78] Bengtsson A., Henriksson K.G. (1989). The muscle in fibromyalgia: A review of Swedish studies. J. Rheumatol..

[79] Oaklander A.L., Herzog Z.D., Downs H.M., Klein M.M. (2013). Objective evidence that small fiber polyneuropathy underlies some illnesses currently labeled as fibromyalgia. Pain.

[80] Levine T.D., Saperstein D.S., Levine A., Hackshaw K., Lawson V. (2016). Small fiber neuropathy in patients meeting diagnostic criteria for fibromyalgia. J. Neurol. Disord..

[81] Lawson V.H., Grewal J., Hackshaw K.V., Mongiovi P.C., Stino A.M. (2018). Fibromyalgia syndrome and small fiber, early or mild sensory polyneuropathy. Muscle Nerve.

[82] Wallace D.J., Gavin I.M., Karpenko O., Barkhordar F., Gillis B.S. (2015). Cytokine and chemokine profiles in fibromyalgia, rheumatoid arthritis and systemic lupus erythematosus: A potentially useful tool in differential diagnosis. Rheum. Int..

[83] Hackshaw K.V., Rodriguez-Saona L., Plans M., Bell L.N., Buffington C.A.T. (2013). A bloodspot-based diagnostic test for fibromyalgia syndrome and related disorders. Analyst.

[84] Diem M., Griffiths P.R., Chalmers J.M. (2008). Vibrational Spectroscopy for Medical Diagnosis.

[85] Kendall C., Isabelle M., Bazant-Hegemark F., Hutchings J., Orr L., Babrah J., Baker R., Stone N. (2009). Vibrational spectroscopy: A clinical tool for cancer diagnostics. Analyst.

[86] Eikje N.S., Aizawa K., Ozaki Y. (2005). Vibrational spectroscopy for molecular characterization and diagnosis of benign, premalignant and malignant skin tumours. Biotechnol. Annu. Rev..

[87] Hackshaw K.V., Miller J.S., Aykas D.P., Rodriguez-Saona L. (2020). Vibrational spectroscopy for identification of metabolites in biologic samples. Molecules.

[88] Miller J.S., Rodriguez-Saona L., Hackshaw K.V. (2020). Metabolomics in central sensitivity syndromes. Metabolites.

[89] Malatji B.G., Meyer H., Mason S., Engelke U.F.H., Wevers R.A., Reenen M., Reinecke C.J. (2017). A diagnostic biomarker profile for fibromyalgia syndrome based on an NMR metabolomics study of selected patients and controls. BMC Neurol..

[90] Hackshaw K.V., Aykas D.P., Sigurdson G.T., Pujolras M.P., Madiai F., Yu L., Buffington C., Giusti M.M., Rodriguez-Saona L. (2019). Metabolic fingerprinting for diagnosis of fibromyalgia and other rheumatologic disorders. J. Biol. Chem..

[91] Sales Passos J.O., Dos Santos Alves M.V., Morais C.L.M., Martin F.L., Cavalcante A.F., Moura Lemos T.M.A., Moura S., Freitas D.L.D., Medeiros Mariz J.V., Carvalho J.L. (2020). Spectrochemical analysis in blood plasma combined with subsequent chemometrics for fibromyalgia detection. Sci. Rep..

[92] Ayvaz H., Rodriguez-Saona L.E. (2015). Application of handheld and portable spectrometers for screening acrylamide content in commercial potato chips. Food Chem..

[93] Raffaeli W., Malafoglia V., Bonci A., Tenti M., Ilari S., Gremigni P., Iannuccelli C., Gioia C., Di Franco M., Mollace V. (2020). Identification of MOR-positive B cell as possible innovative biomarker (mu lympho-marker) for chronic pain diagnosis in patients with fibromyalgia and osteoarthritis diseases. Int. J. Mol. Sci..

